# Effects of Electron Beam Irradiation on the Mechanical, Thermal, and Surface Properties of Some EPDM/Butyl Rubber Composites

**DOI:** 10.3390/polym10111206

**Published:** 2018-10-30

**Authors:** Maria Daniela Stelescu, Anton Airinei, Elena Manaila, Gabriela Craciun, Nicusor Fifere, Cristian Varganici, Daniela Pamfil, Florica Doroftei

**Affiliations:** 1 National Research and Development Institute for Textile and Leather, Leather and Footwear Institute, 93 Ion Minulescu Street, Bucharest 031215, Romania; dmstelescu@yahoo.com; 2 Petru Poni Institute of Macromolecular Chemistry, 41A Grigore Ghica Voda Alley, Iasi 700487, Romania; fifere.nicusor@icmpp.ro (N.F.); varganici.cristian@icmpp.ro (C.V.); pamfil.daniela@icmpp.ro (D.P.); florica.doroftei@icmpp.ro (F.D.); 3 National Institute for Lasers, Plasma and Radiation Physics, 409 Atomistilor Street, Magurele 077125, Romania; elena.manaila@inflpr.ro (E.M.); gabriela.craciun@inflpr.ro (G.C.)

**Keywords:** EPDM, electron beam irradiation, butyl rubbers, mechanical properties, thermal stability, crosslinking, contact angle

## Abstract

The effects of electron beam irradiation on the properties of ethylene propylene diene monomer (EPDM)/butyl rubber composites in presence of a polyfunctional monomer were investigated by means of differential scanning calorimetry (DSC), thermal analysis, scanning electron microscopy (SEM), attenuated total reflection absorption infrared spectroscopy (ATR-IR), and mechanical and surface energy measurements. The samples were exposed over a wide range of irradiation doses (20–150 kGy). The EPDM matrix was modified with butyl rubber, chlorobutyl rubber, and bromobutyl rubber. The gel content and crosslink density were found to increase with the electron beam irradiation dose. The values of the hardness and modulus increased gradually with the irradiation dose, while the tensile strength and elongation at break decreased with increasing irradiation dose. The EPDM/butyl rubber composites presented a higher thermal stability compared to the initial EPDM sample. The incorporation of butyl rubbers into the EPDM matrix led to an increase in material hydrophobicity. A similar trend was observed when the irradiation dose increased. The greatest change in the surface free energy and the contact angles occurs at an irradiation dose of 20 kGy. The Charlesby–Pinner plots prove the tendency to crosslinking as the irradiation dose increases.

## 1. Introduction

Polymerization of ethylene, propylene, and a nonconjugated diene results in obtaining ethylene propylene diene monomer (EPDM) rubber having a saturated polymer backbone and a very low content of unsaturation in the side groups. Among elastomers, EPDM exhibits excellent thermal stability and high resistance to heat, ozone, radiation temperature, ageing, etc. [1,2,3,4]. In addition, due to its nonpolar character, EPDM has superior electric resistivity, particular retention properties (even after ageing), and high resistance against polar solvents such as water, acids, alkalies, ketones, or alcohols [3,4]. Due to the special electrical properties, ageing resistance, and elevated temperature resistance, EPDM elastomers can be utilized as electrical insulating materials, automotive profiles, electrical cables, roofing materials, window gaskets, or in different nuclear applications [1,5,6].

To improve some physical and mechanical characteristics of EPDM rubber and to obtain different technical goods with particular properties, EPDM can be associated with other elastomers, such as butyl rubber (IIR) or halobutyls (chlorobutyl rubber (Cl–IIR) and bromobutyl rubber (Br–IIR)). Butyl rubber has a low gas and moisture permeability, good weathering resistance, excellent resistance to oxygen and ozone attack, and chemical stability to a great number of organic and inorganic media, but it has a limited resistance to thermal ageing. Due to the absence of double bonds in the backbone, butyl rubber is stable in diluted solutions of acids or alkalies, as well as to prolonged heat exposure. Also, butyl rubber, due to its excellent properties, has found applications as materials utilized in high-pressure conditions and to damp shocks and vibrations [5,7]. The specific working range for products based on butyl rubber is from −50 to 120 °C. The physical and compositional properties of butyl rubbers vary as a function of molecular weight, unsaturation, and branching degree, and these polymers exhibit a very low crystallization tendency. The chlorination and bromination process of butyl rubber leads to the halogenation of isoprene units in butyl rubber. Chlorobutyl rubbers present a low gas and moisture permeability, good resistance to weathering and hydrocarbon solvents, and high thermal stability [5,8]. EPDM has better mechanical properties than the butyl/halogenated butyl rubbers. Because of the high thermal stability of butyl rubber/halogenated butyl rubber, their blends with EPDM would be more attractive.

Butyl rubber or halobutyl rubber, due to its low air permeability, can generate some problems in processing because during vulcanization some blisters can be formed on the composite surface [9]. Such problems may be avoided by using electron beam crosslinking of the EPDM/IIR, EPDM/Hal–IIR blends at room temperature. The electron beam vulcanization leads to superior results compared to the conventional crosslinking systems which require high temperature, and it eliminates the utilization of sulfur and other crosslinking agents which generate toxic compounds.

Irradiation with accelerated electron beams presents some advantages over conventional processing because the crosslinking of EPDM is made at room temperature, thus avoiding polymer degradation and oxidative degeneration, as observed in classical vulcanization. The crosslinking via electron beam irradiation is an alternative to classical vulcanization to obtain materials with high crosslinking degree; high tensile strength; good resistance to compression; extremely high resistance to oils, lubricants, and greases; short processing times; and very low material wastes [4,10,11]. The crosslinking of EPDM rubber by electron beam irradiation—although it is a procedure that requires greater production costs—can be applied at industrial level by reducing the irradiation dose using polyfunctional monomers. In this way, rubber degradation can be avoided [12,13,14].

The aim of the paper was the modification of the surface properties and the improvement of the thermal stability of some elastomeric materials based on EPDM obtained by blending with butyl rubber/halobutyl rubber and by processing with accelerated electron beams. The effect of accelerated electron beam irradiation dose on the mechanical, thermal, and morphological properties was investigated compared to an EPDM sample without butyl rubber. The above-mentioned properties were correlated with IR spectral data, crosslinking density, and contact angle measurements. Although the irradiation of EPDM was applied, we mention that the processing by electron beams of EPDM materials is a convenient method to modify some structural or surface properties at room temperature without other ingredients. We obtained materials with good stability to electron beam irradiation and with modified surface properties when the samples become more hydrophobic; this characteristic was maintained regardless of irradiation dose.

## 2. Experimental

### 2.1. Materials

EPDM rubber (Nordel 4760) having 70% ethylene content, 4.9% 5-ethylene norbornene as the diene, and Mooney viscosity [ML (1 + 4) at 120 °C] of 70, density 0.88 g/cm^3^, and crystallinity degree 10% was procured from Dow Chemical Company (Michigan, MI, USA). Butyl rubbers Butyl 268 (Mooney viscosity [ML (1 + 8) at 125 °C] 51, unsaturation degree 1.70 mol %), Chlorobutyl HT1066 (Mooney viscosity [ML (1 + 8) at 125 °C] 38, unsaturation degree 1.26 mol %), and Bromobutyl 2222 (Mooney viscosity [ML (1 + 8) at 125 °C] 38, unsaturation degree 1.03 mol %) were supplied by Exxon Mobil Chemicals (Machelen, Belgium). Trimethylolpropane trimethacrylate, Luvomaxx TMPT DL 75 (Hamburg, Germany) (ash content 22%, pH 9.2, density 1.36 g/cm^3^, active ingredient 75.53%), was used as the polyfunctional monomer. Pentaerythritol tetrakis [3-(3,5-ditert-butyl-4-hydroxy)propionate] (Irganox 1010) was obtained from BASF Schweiz (Basel, Switzerland) (melting point of 40 °C, 98% active ingredient) and was utilized as the antioxidant.

### 2.2. Methods

EPDM/butyl rubber composites were prepared by melt blending in a Brabender Plasticorder 828703 V230 (Duisburg, Germany) at high temperature (160–190 °C) for 10 min blending time and at a 30–150 rpm rotor speed. This procedure improves the dispersion degree of butyl rubber/halobutyl rubber in the EPDM matrix, assuring an efficient incorporation of butyl rubber in EPDM. The formulations of the prepared rubbery samples and their designations are given in Table 1. The homogenization of blends was performed on a laboratory electrically heated roller mill with the following working parameters: temperature 60–80 °C, friction 1.1, total mixing time 7 min, and 2–4 mm thick rubber sheets were obtained. Test specimens were made by compression molding using a laboratory hydraulic press (Fortune Presses, model TP600-Fontyne Grotnes, Vlardingen, The Netherlands) at a temperature of 160 °C and pressing force of 300 kN, preheating time of 1 min, and molding time of 8 min at 35 °C to obtain approximately 2 mm thick sheets.

The samples prepared as described above were packed in polyethylene film in order to avoid sticking and were then irradiated at 20, 50, 100, and 150 kGy using an ALID7 electron beam accelerator. Irradiations were performed in atmospheric conditions at room temperature (25 °C). The electron beam accelerator was a travelling wave type operating at a wavelength of 10 cm. The accelerating structure was a disk-loaded tube operating in the π/2 mode. The optimum values of the electron beam, peak current (I_EB_), and electron beam energy (E_EB_) to produce a maximum output power (P_EB_) for a fixed pulse duration (τ_EB_) and repetition frequency (f_EB_) were as follows: E_EB_ = 5.5 MeV, I_EB_ = 130 mA, P_EB_ = 670 W, f_EB_ = 250 Hz, and τ_EB_ = 3.75 μs. The radiation dosimetry was assured using a graphite calorimeter, which is a primary standard used for the calibration of the absorbed dose measurements for high-energy electron beam treatments. Rubber sheets in rectangular shapes of 100 mm × 100 mm × 2 mm were irradiated at radiation doses of 20, 50, 100, and 150 kGy.

The tensile strength determinations were carried out on a Schopper strength tester employing a testing speed of 460 mm/min, according to ISO 37/2017, on dumbbell-shaped specimens. The hardness of rubber composites was measured according to ISO 7619-1/2011, using a hardness tester. The unit of hardness was expressed in Shore A. The elasticity measurement was performed using a Schob test instrument on 6 mm thick samples, according to ISO 4662/2017.

The gel content of electron beam crosslinked EPDM samples was estimated by a solvent extraction method using toluene as the solvent. Samples of 1.0 × 1.0 cm were initially weighed (*w*_1_) and immersed in toluene for 72 h in order to realize the equilibrium swelling condition. Then, the samples were dried in air for six days at room temperature and were weighed again (*w*_3_). The gel fraction was calculated by the relation

(1) $$\mathrm{Gel}\;\mathrm{fraction}=\frac{w_{3}}{w_{1}}\times 100$$

where *w*_3_ and *w*_1_ denote the weight of the dried sample after toluene extraction and the initial weight before extraction [4,15]. The obtained results represent the average of five specimens.

The crosslinking density (*ν*) of the EPDM/butyl rubber samples was determined from swelling data in toluene by applying the modified Flory–Rehner relation for tetrafunctional networks. Swelling measurements were carried out in a 500 mL dark-colored bottle equipped with a stopper. The volume of toluene used in sample immersion was 350 mL, according to ISO 1817:2015. With this in view, specimens of 2 mm thickness and initial weight *m*_1_ were immersed in toluene for 72 h at room temperature. The swollen samples were removed from toluene, dried in air to eliminate excess solvent, and weighed (*m*_2_). The solvent traces were eliminated by drying in air for six days at room temperature and the samples were then weighed once again (*m*_3_). The volume fraction of rubber in the swollen network, *ν*_2*m*_, was determined from the swelling ratio according to the expression

(2) $$\nu_{2m}=\frac{1}{1+G}$$

where

(3) $$G=\left(m_{2}-m_{3}\right)/m_{3}\cdot \rho_{r}/\rho_{s}$$

and *ρ_r_*, *ρ_s_* are the densities of the EPDM/butyl rubber sample and toluene (0.865 g/cm^3^).

The Flory–Rehner equation was applied for the determination of the crosslinking degree (*ν*) [16]:

(4) $$\nu =-\frac{\ln\left(1-\nu_{2m}\right)+\nu_{2m}+\chi_{12}\nu_{2m}^{2}}{V_{1}\left(\nu_{2m}^{1/3}-2/\Phi \cdot \nu_{2m}\right)}$$

where *ν* is the crosslinking density, *ν*_2*m*_ is the volume fraction of rubber in the solvent swollen sample, *χ*_12_ denotes the Flory–Huggins polymer–solvent interaction parameter, Φ = 4 is the crosslinking functionality, and *V*_1_ denotes the molar volume of toluene (106.5 cm^3^/mol).

FTIR absorption spectra were recorded on a PERKIN ELMER FT-IR Spectrum 100 spectrometer (Shelton, CT, USA) in ATR mode with a diamond/ZnSe crystal. A total of ten scans were performed for each sample with a resolution of 4 cm^−1^. The samples were scanned from 4000 to 600 cm^−1^.

The thermal degradation experiments were performed on a STA 449F1 Jupiter device (Netzsch, Germany), using 10 mg of each sample that was heated from 30 to 700 °C under a nitrogen flow rate of 50 mL/min in an open Al_2_O_3_ crucible.

Differential scanning calorimetry (DSC) measurements were conducted on a DSC 200 F3 Maia instrument (Netzsch, Germany) in nitrogen atmosphere at a flow rate of 50 mL/min at heating and cooling rates of 10 °C/min and −10 °C/min, respectively. A mass of 8 mg of sample was heated in pierced and sealed-shut aluminium crucibles. The temperature against heat flow was recorded. The baseline was obtained by scanning the temperature range of the experiments with an empty pan. The instrument was calibrated with indium at various heating rates according to standard procedures. The glass transition temperature (T_g_), melting temperature (T_m_), crystallization temperature (T_c_), melting heat of fusion (ΔH_m_), and crystallization enthalpy (ΔH_cr_) were estimated from DSC curves.

The static contact angle measurements were performed using a CAM-200 instrument (KSV Instruments, Helsinki, Finland) by the sessile drop method, at room temperature, after placing 1 μL drop of liquid on the sample surface. Water, formamide, and diiodomethane were used as probe liquids. For each liquid, five measurements at different locations on the sample surface were made. The average values were recorded for further evaluation of the results.

The morphology of the cross-section samples was examined with a Quanta 200 Scanning Electron Microscope (FEI, Brno, The Czech Republic), operating at 20 kV in low vacuum mode using a large field secondary electron detector.

## 3. Results and Discussion

### 3.1. Mechanical Properties

**Figure 1:**
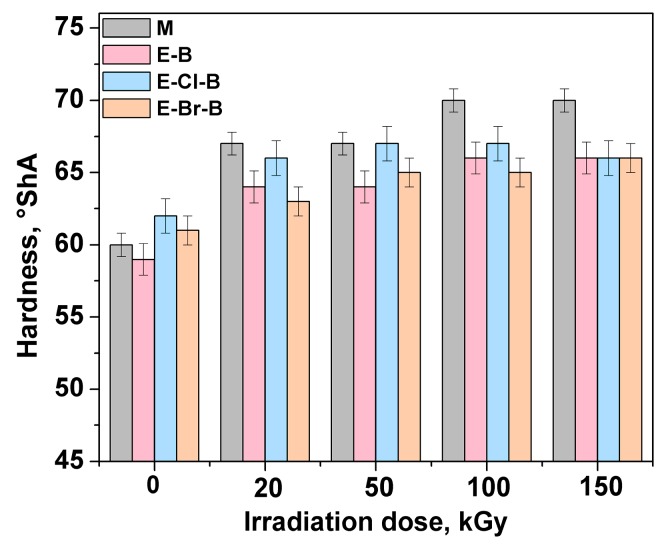
Hardness versus electron beam irradiation dose for EPDM/butyl rubber samples.

**Figure 2:**
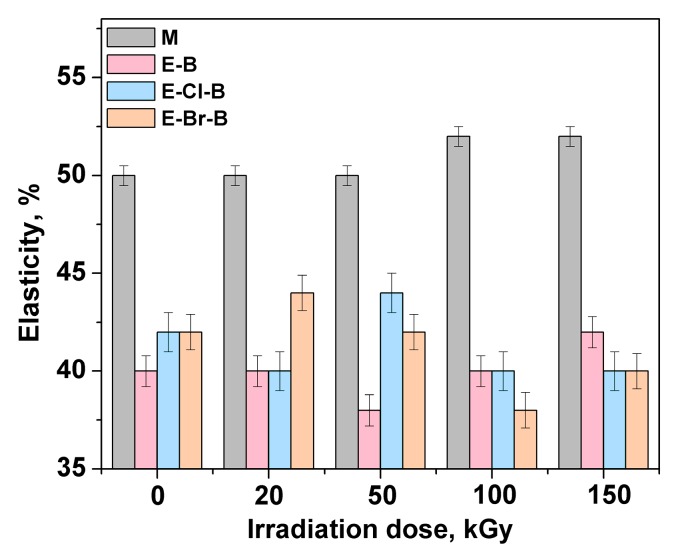
Elasticity of EPDM/butyl rubber composites as a function of irradiation dose.

**Figure 3:**
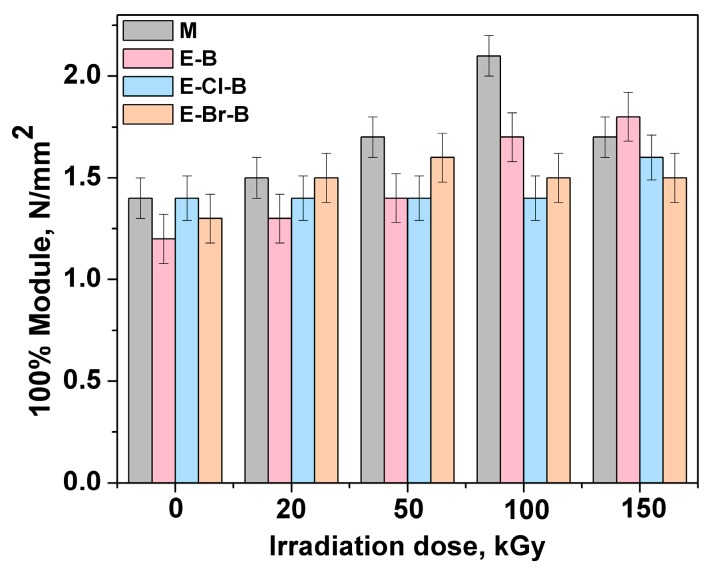
Variation of 100% modulus with electron beam irradiation dose.

**Figure 4:**
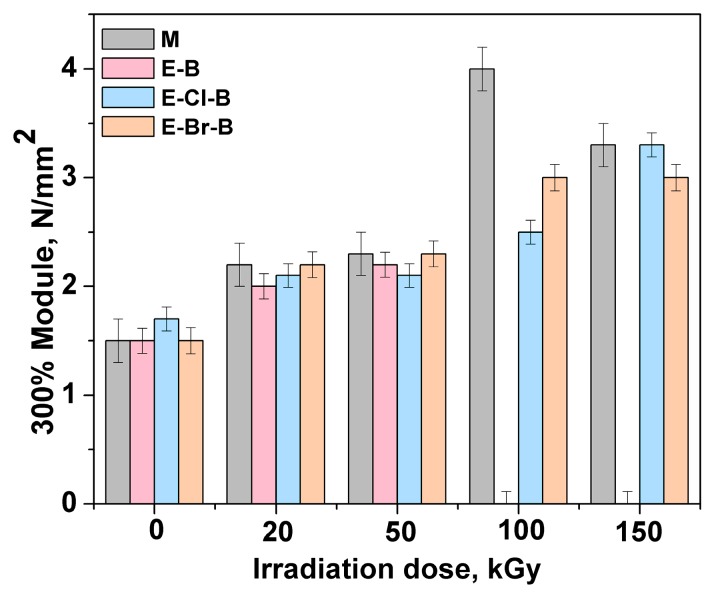
Variation of 300% modulus of EPDM/butyl rubber composites with irradiation dose.

**Figure 5:**
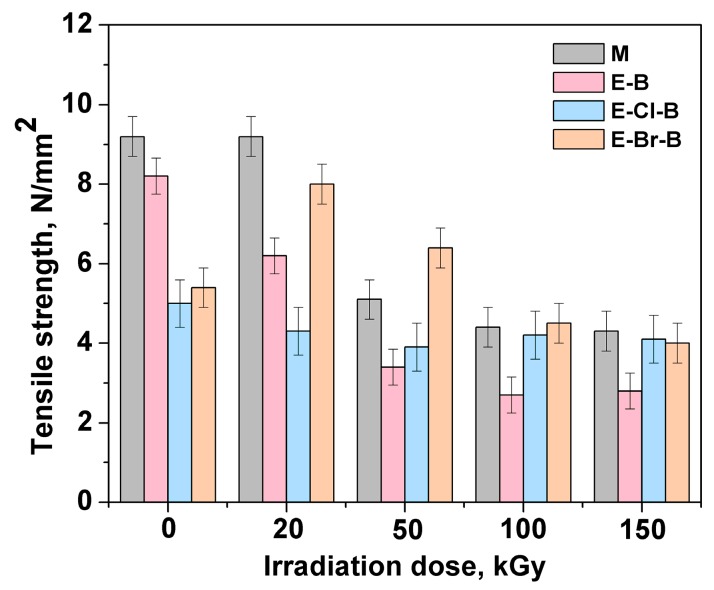
Tensile strength of EPDM/butyl rubber composites as a function of irradiation dose.

**Figure 6:**
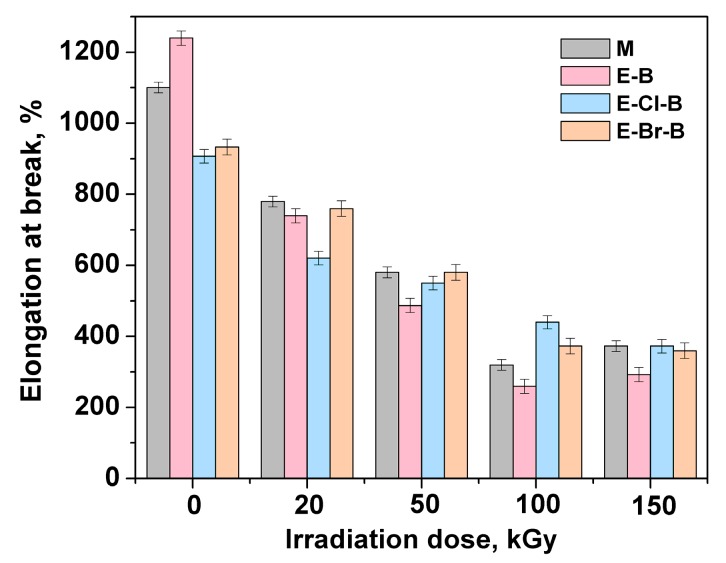
Variation of elongation at break of EPDM/butyl rubber composites with irradiation dose.

**Figure 7:**
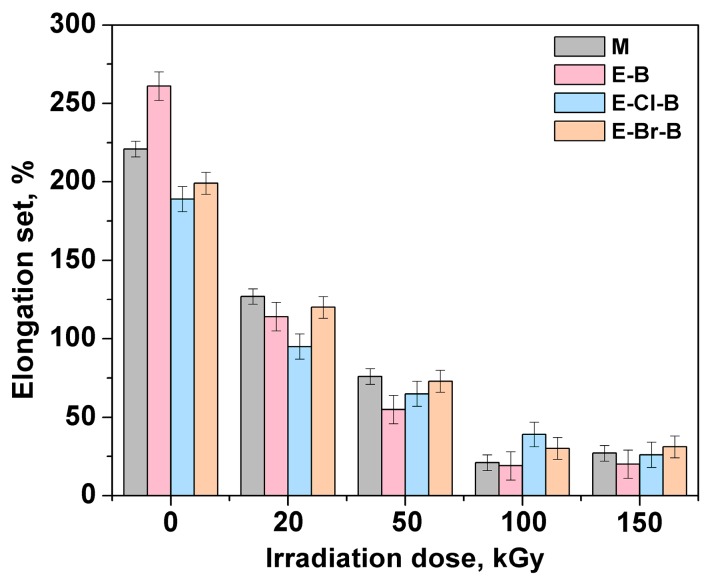
Effect of irradiation dose on elongation set.

The mechanical properties, such as tensile strength, hardness, elasticity, elongation at break, modulus, and elongation set, were investigated as a function of irradiation dose; the corresponding data are summarized in Figure 1, Figure 2, Figure 3, Figure 4, Figure 5, Figure 6 and Figure 7. The results indicated that the hardness, 100% modulus, and 300% modulus increased with irradiation dose increase (Figure 1, Figure 3 and Figure 4). By irradiation with accelerated electron beams, two processes can occur concomitantly, namely, crosslinking and chain scission. The first process appears frequently at lower electron beam irradiation doses, up to 150 kGy, while the second process leads to C–C bonds cleavage at higher doses [17,18]. The crosslinking determines the increase in tensile strength but reduces elongation, while the scission process leads to both tensile strength and elongation decrease. Because EPDM contains an aliphatic chain which has low resistance to the influence of ionizing radiation, at higher irradiation doses the degradation can be predominant as compared to crosslinking [18,19]. From Figure 1, it may be seen that the control sample (M) attained the greatest values of hardness, relatively higher than those of irradiated composites. The increase in hardness of the irradiated composites can be determined by the crosslinking reactions occurring in the system. Also, the 100% modulus and 300% modulus increased slightly with irradiation dose (Figure 3 and Figure 4) as a result of the crosslinking due to electron beam irradiation. As expected, the control sample (M) has the lowest value of 300% modulus, and the modulus values then increase as the irradiation dose increases, with the samples irradiated at higher doses (100 and 150 kGy) having the highest values of 300% modulus. This improvement of the modulus compared to nonirradiated samples may be the manifestation of crosslinking in composites and a better interfacial bonding between EPDM and butyl rubber.

The tensile characteristics depend on the crosslinking density, molecular imperfection, or reduced molecular weight due to the degradation, as well as on the compatibility between the two elastomers from composition [20]. The EPDM rubber used in this work has high ethylene content and it is a crystalline material (crystallinity degree of 10%). The crystallization which can occur acts as a spurious crosslinking and the rubber composition will become rigid. As shown from Figure 1, Figure 2, Figure 3, Figure 4, Figure 5, Figure 6 and Figure 7, the electron beam irradiation and the incorporation of butyl/halogenated rubber can lead to a decrease of the tensile strength. The elasticity, tensile strength, elongation at break, and elongation set present a relatively faster rate of decrease in their values as a function of irradiation dose, the smallest values being obtained at higher doses (Figure 2, Figure 5 and Figure 7). The tensile strength and elongation at break for EPDM rubber increase up to a certain value of crosslinking density; further increase of crosslink density leads to the decrease of these values. The value of irradiation dose for which the tensile strength is optimum depends on the sample composition, as can be seen from Figure 5. However, the values of tensile strength of EPDM are greater than those of butyl rubber or halobutyl rubber [21]. The tensile strength has been found to be higher for composites containing Br-butyl rubber due to the strong polar character of the bromobutyl moiety. The lower values of some mechanical characteristics of EPDM/chlorobutyl rubber are due to the ability of chlorine radicals to substitute hydrogens from the main chain. If the crosslink density is too high, the chain segment mobility becomes more restricted and a decrease in tensile strength values can occur. The incorporation of butyl or halobutyl rubber into the EPDM matrix gives rise to a significant decrease in elasticity, elongation at break, and elongation set as the irradiation dose increases (Figure 2, Figure 6 and Figure 7). The elongation at break decreases continuously upon electron beam irradiation due to the crosslink formation which restricts the movement of polymer chains. The network structure by radiation crosslinking prevents the structural reorganization during applied force, leading to the decrease of the elongation at break. Also, the increase of crosslink density at higher irradiation doses determines the decrease of polymer chains mobility, and the elongation at break will be lower [22].

### 3.2. Gel Fraction and Crosslink Density

In order to estimate the crosslink density, the Flory–Huggins interaction parameter between polymer and solvent was calculated using Equation (5), according to Blanks and Prausnitz [23]:

(5) $$\chi =\chi_{s}-\chi_{H}=\chi_{s}+\frac{\nu_{\mathrm{ms}}}{RT}{\left(\delta_{s}-\delta_{p}\right)}^{2}$$

where *χ*_s_ is the entropic contribution of this parameter (usually 0.34) [21]; *χ*_H_ is the enthalpic contribution, determined from the molar volume of solvent, *ν*_ms_; *R* is the universal constant of gases; *T* denotes the absolute temperature; and *δ*_s_ and *δ*_p_ represent the Hildebrand solubility parameters of polymer and solvent (18.2 (MPa)^1/2^ for toluene) [24], respectively. The solubility parameter of the polymer was computed using the semiempirical group additivity theory based on the models proposed by Small [25], Hoy [26], and Van Krevelen [26]. The determination of the Hildebrand solubility parameter requires the value of the molar attraction constant, *F*_i_, for each chemical group from the polymer repeating unit, according to the relation

(6) $$\delta_{p}=\frac{\sum F_{i}}{V}$$

where *V* is the molar volume of the polymer (cm^3^ mol^−1^).

The molar volumes of EPDM, EPDM/IIR, EPDM/Cl–IIR, and EPDM/Br–IIR are as follows: 301.6417, 438.1559, 476.0076, and 524.8548 cm^3^/mol, respectively. The chemical groups from the repeating units of the above-mentioned polymers, as well as their molar attraction constants, according to the Small, Hoy, and Krevelen models [24], are given in Table 2. The molar attraction constants for the repeating units, Σ*F_i_*, of these polymers are illustrated in Table 3. The values of solubility parameter estimated the by Small, Hoy, and Van Krevelen methods, as well as the Flory–Huggins interaction parameter, are listed in Table 4. To compute the crosslink density, the values of interaction parameters estimated with the Van Krevelen model were utilized, being in good agreement with *χ* values for the EPDM–toluene system (*χ* = 0.49) [27].

**Figure 8:**
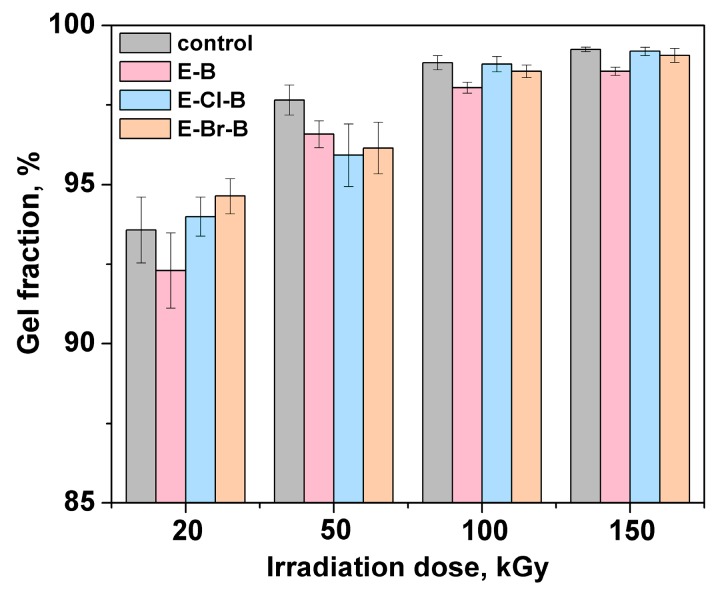
Evolution of gel fraction for EPDM rubber as a function of irradiation dose.

**Figure 9:**
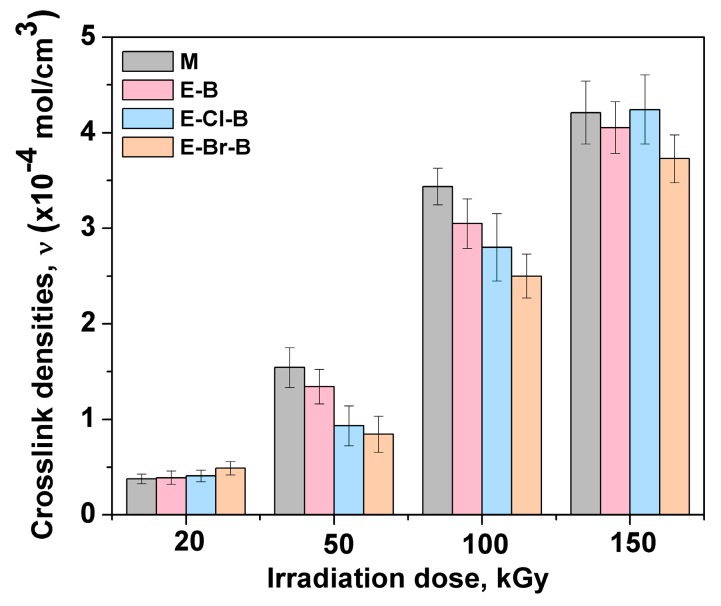
Variation of crosslink density with irradiation dose.

It can be seen from Figure 8 and Figure 9 that the gel content and crosslink density increase with increasing irradiation dose for each EPDM composite. As a result of the increase in the crosslinking extent, the gel fraction increases gradually. A maximum gel content of 99% was observed for E–Cl–B and E–Br–B samples at an irradiation dose of 150 kGy. The incorporation of butyl rubber or halobutyl rubber into the EPDM matrix leads to the decrease of crosslink density for the same irradiation dose (Figure 9). The chlorinated and brominated butyl rubbers (Cl–IIR and Br–IIR) are obtained by the halogenation reaction of the isoprene units from butyl rubber. By breaking the weaker C–Hal bonds, organic radicals can be generated from the halogenated isoprene moieties. A higher breaking yield under electron beam irradiation was observed in chlorinated butyl rubber as compared to the substitution of hydrogen by chlorine atoms leading to the polymer chain breaking in butyl rubber [13,28]. It was observed that the good values of tensile strength correspond to a crosslink degree of around 1 × 10^−4^ mol/cm^2^. These results are in agreement with data reported by other authors showing that the vulcanizates based on butyl and halobutyl rubber exhibit approximately the same value of crosslink density [29], as well as our data previously obtained for EPDM rubber crosslinked by electron beam irradiation in the presence of a polyfunctional monomer—trimethylolpropane trimethacrylate (TMPT) [30].

**Scheme 1:**
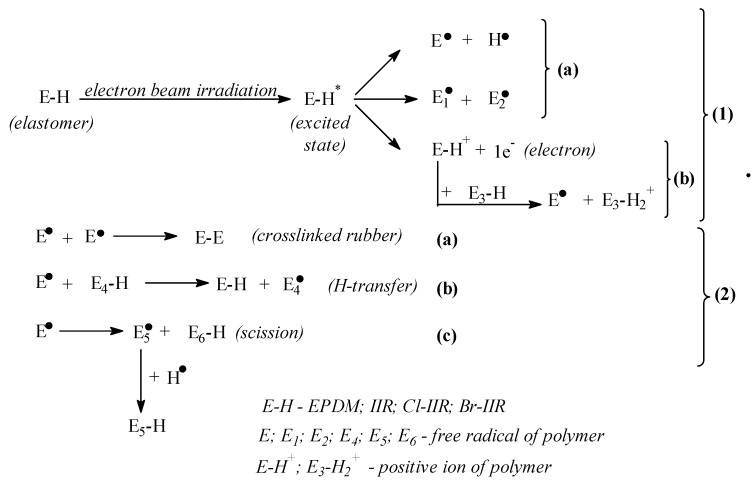
Crosslinking and scission reactions occurring in elastomeric composites during accelerated electron beam irradiation.

The irradiation with accelerated electron beams provides excited molecules by energy transfer or indirectly by the neutralization of resulting ions. If the excited state has adequate energy, the breaking of covalent bonds takes place and two radical fragments are formed (Scheme 1, reactions 1a). An ionization process can be the second effect due to electron beam irradiation (Scheme 1, reactions 1b). The free radicals formed during irradiation can initiate polymerization, grafting, crosslinking, or degradation reactions depending on the irradiation dose. Here we utilized higher irradiation doses, and the crosslinking and scission reactions can occur (Scheme 1, reactions 2).

**Figure 10:**
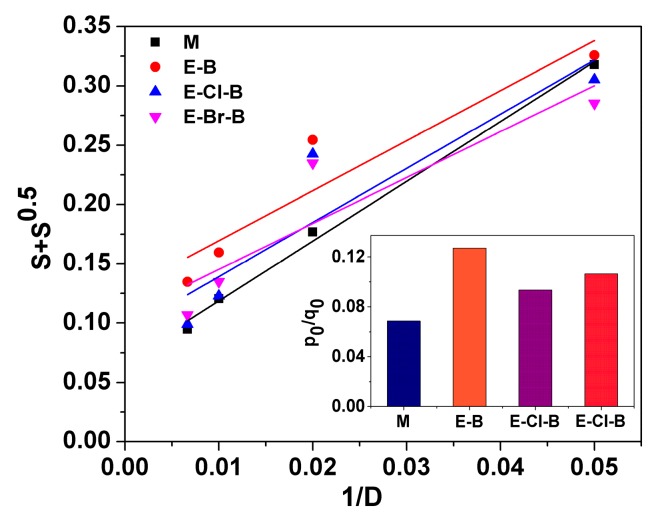
Charlesby–Pinner plots for EPDM/butyl rubber composites at various irradiation doses.

Using the Charlesby–Pinner equation it is possible to estimate the ratio of crosslinking and chain scission [31,32]:

(7) $$S+\sqrt{S}=\frac{p_{0}}{q_{0}}+\frac{1}{\alpha P_{n}D}$$

where *D* is the irradiation dose in kGy, *S* is the sol fraction, *p*_0_ represents the average number of chain scissions per monomer unit per dose unit, *q*_0_ is the crosslinking density per dose unit, *P_n_* is the numbered average degree of polymerization, and α = *q*_0_/2. The plots of (*S* + *S*^1/2^) as a function of reciprocal irradiation dose for EPDM/butyl rubber composites are given in Figure 10. As shown in Figure 10 the values of the *p*_0_/*q*_0_ ratio are smaller than 1, suggesting a significant tendency to crosslinking as the irradiation dose increases. The sample M, without butyl rubber and with the lowest value of *p*_0_/*q*_0_, is most efficiently crosslinked on electron beam irradiation, in agreement with previous reports [33]. The crosslinking extent decreases in the presence of butyl rubber or halobutyl rubbers, and the *p*_0_/*q*_0_ ratio increases (Figure 10) because the elastomers can undergo some degradation processes induced by electron beam irradiation. In the case of butyl rubber, the crosslinking process can occur by isoprene units, but the scission reactions of isobutylene units can be present. For halobutyl rubber the radicals can be provided by halogenated isoprene bonds [13,34].

### 3.3. FTIR Analysis

**Figure 11:**
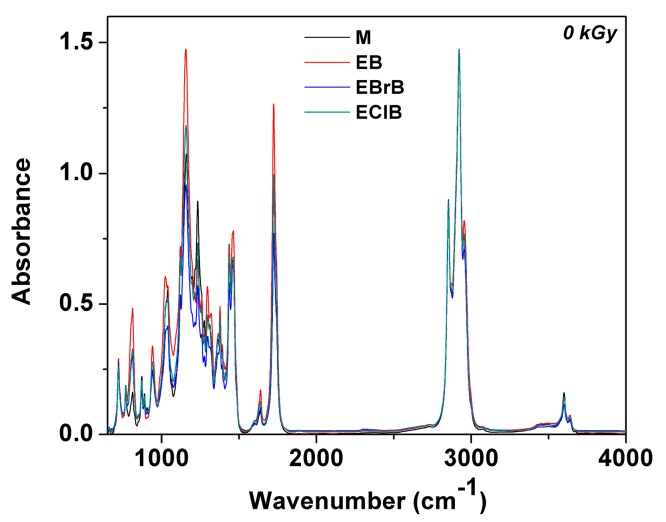
FTIR spectra of nonirradiated EPDM/butyl rubber composites.

**Figure 12:**
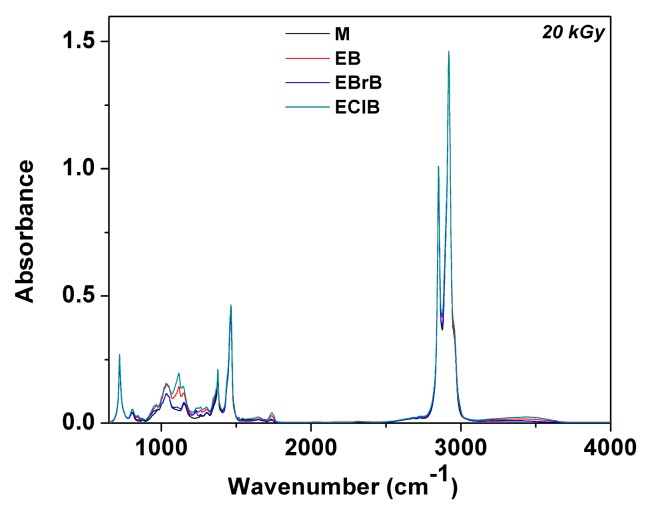
FTIR spectra of electron-beam-exposed EPDM/butyl rubber composites (20 kGy).

**Figure 13:**
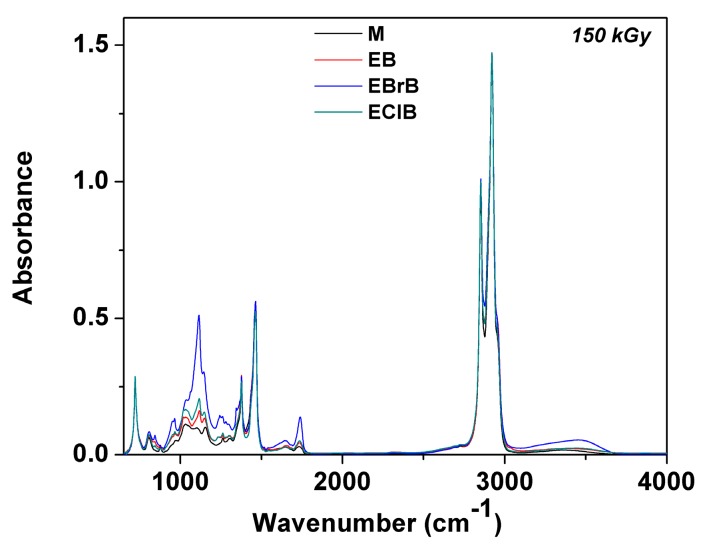
FTIR spectra of EPDM/butyl rubber composites irradiated at 150 kGy.

Analysis of IR absorption spectra of EPDM/butyl rubber composites, shown in Figure 11, Figure 12 and Figure 13, reveals the IR absorption bands characteristic of EPDM: 1462 cm^−1^ assigned to –CH_2_ scissoring vibrations, 1376 cm^−1^ to C–H bending vibration of the –CH_3_ group, and 722 cm^−1^ attributed to –CH_2_ rocking vibrations, due to the ethylene sequences in the polymer backbone [35,36,37]. Also, the absorption bands at around 2922 and 2852 cm^−1^ are typical for EPDM rubber and they correspond to the saturated hydrocarbon backbone, namely, to C–H symmetric and asymmetric stretching vibrations, respectively. During electron beam irradiation of EPDM samples, the absorption bands around 1725 and 1638 cm^−1^ assigned to the >C=O stretching vibration and >C=C< stretching vibration from TMPT [38] decrease strongly in intensity due to the crosslinking, in agreement with the observations of Fujimoto [39,40]. The significant modifications of the absorption bands during electron beam irradiations appear at lower irradiation doses, at about 20 kGy (Figure 12). Irradiation with higher doses does not change the spectral profile (Figure 13) and the SEM data show the same result. The increase of crosslink density can be attributed not only to the presence of double bonds but also to the formation of macroradicals during irradiation which by recombination reactions determine the material structuration. Also, free radicals can be generated by TMPT molecules under electron beam irradiation, leading to the grafting of TMPT onto rubber. The absorption bands due to TMPT grafted onto EPDM decrease gradually in intensity, probably due to the vulcanization process of EPDM in the presence of the polyfunctional monomer, and the double bonds from TMPT are consumed during irradiation. It may be noticed that the small modifications in intensity of the absorption bands of EPDM/butyl rubber composites at higher irradiation doses (Figure 13) are in agreement with the variation of crosslink density at higher electron beam irradiation doses for both the control and butyl-rubber-modified EPDM samples (Figure 9). However, the presence of absorption bands at around 2922, 1452, 1376, and 720 cm^−1^ proves the fact that the polymer backbone of the EPDM sample is not destroyed by electron beam irradiation.

### 3.4. Thermal Behavior

**Figure 14:**
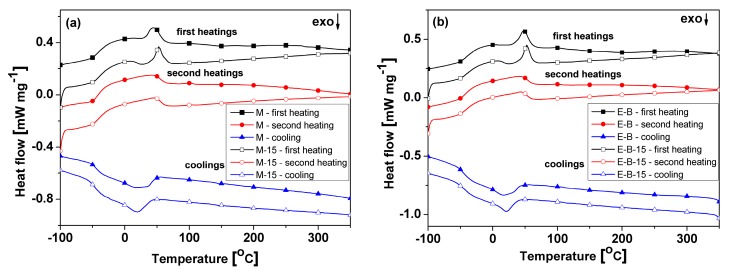

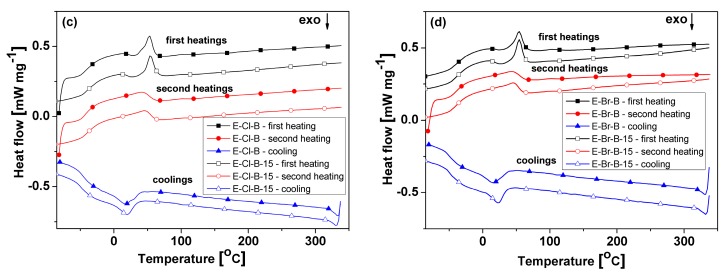
DSC thermograms of nonirradiated samples and those irradiated at 150 kGy: (**a**) M; (**b**) E–B; (**c**) E–Cl–B; (**d**) E–Br–B.

The DSC method was employed to estimate the glass transition temperatures of EPDM/butyl rubber samples. Representative DSC thermograms of nonirradiated samples and those irradiated with electron beams at 150 kGy are depicted in Figure 14. Since no significant differences in thermal behavior patterns were observed at higher doses of electron beam irradiation, comparative DSC scans of initial structures and those irradiated at 150 kGy were analyzed (Figure 14). Some data extracted from the DSC thermograms are given in Table 5.

The analysis of the thermograms indicates that the glass transition temperature (T_g_) of nonirradiated EPDM composites ranges between −35 and −40 °C and a melting profile (T_m_) can be observed with a maximum peak in the range 41–54 °C, for both heating scans, depending on sample composition variation. The presence of a single well-defined T_g_, even after electron beam irradiation, is an indication of good component miscibility [11,41]. The crystallization temperature (T_cr_) varies in the range 13–33 °C. As can be seen in Table 5, the T_g_ values exhibit a general increasing tendency for the irradiated samples, due to decrease of molecular mobility, as a result of crosslinking [6,42]. T_m_ and crystallization temperature (T_cr_) values also generally shifted to higher domains, together with the decrease in melting enthalpy (ΔH_m_) and crystallization enthalpy (ΔH_cr_) after the electron beam irradiation. This observation confirms that the electron beam irradiation generates crosslinking phenomena with the decrease in the crystalline fraction of the studied compounds. Also, while both nonirradiated and irradiated samples E–Cl–B and E–Br–B exhibit cold crystallization transition (T_cc_) on the first heating scans, probably induced by the presence of the more bulky Cl and Br atoms, samples M and E–B show this phenomenon only after the electron beam irradiation and with higher enthalpy (ΔH_cc_) values. This fact may be an indication of the presence of a higher amorphous fraction due to crosslinked longer polymer chains of higher mobility.

Table 6 summarizes some thermal characteristics of the EPDM/butyl rubber composites extracted from the TG data, such as temperatures corresponding to 5 wt %, (T_5%_), 10 wt % (T_10%_), 30 wt % (T_30%_), 50 wt % (T_50%_) mass loss, and to the maximum rate of decomposition (T_max_); mass loss per each thermal decomposition stage (W); and residual mass left at the end of the thermal degradation process (700 °C) (R).

It may be seen from Table 6 that one decomposition stage is observed for all nonirradiated and electron-beam-irradiated EPDM/butyl rubber samples, corresponding to the decomposition of EPDM for sample M and to its mixture with the butyl rubbers in the materials for the other samples. The presence of a single thermal degradation stage is also an indication of the good miscibility between material components, with the samples behaving as single-component systems [43]. The nonirradiated structures start to decompose in the temperature range 410–433 °C (T_5%_), whilst the those electron beam irradiated at 150 kGy thermally degrade at higher temperatures (415–445 °C). The higher thermal degradation temperature domains suggest the occurrence of crosslinking processes during irradiation. This fact is in good agreement with the DSC data. The irradiated samples also exhibit higher T_10%_, T_30%_, T_50%_, and T_max_ values, lower mass loss values (*W*), and higher residue masses at 700 °C, thus confirming crosslinking occurrence (Table 6).

### 3.5. Morphological Characterization

**Figure 15:**
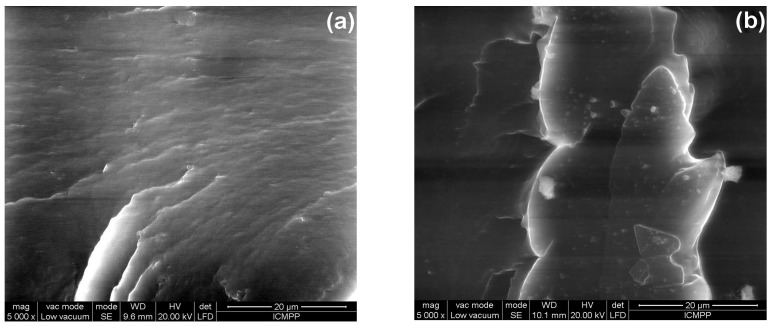

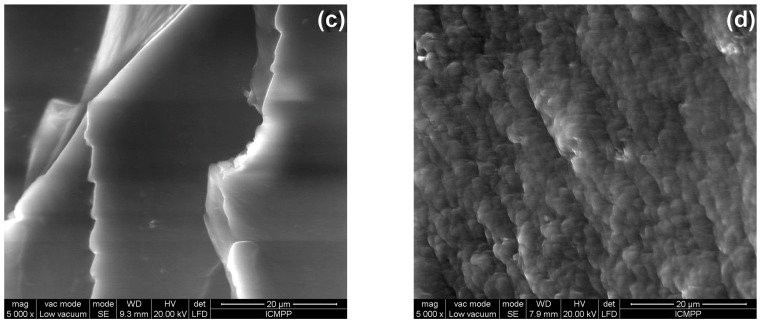
Micrographs of fractured surfaces of EPDM (M (**a**)) and EPDM blends (E–B (**b**); E–Cl–B (**c**); E–Br–B (**d**)) before irradiation (5000× magnification).

Scanning electron microscopy (SEM) was used to investigate fracture surface morphologies of the various EPDM sample compositions. The SEM micrographs of the fractured surfaces of EPDM (M) and EPDM blends (E–B, E–Cl–B, E–Br–B) before irradiation are depicted in Figure 15. In the case of elastomers, it is well known that the way in which fracture occurs can be correlated with their properties. By fracturing, due to the low modulus of elasticity, the elastomers show a typical “wave” morphology. This typical morphology can be observed for all EPDM samples. On the other hand, in the case of sample M one can observe a microstructure with submicron domains that determine a certain roughness of the fracture. The fracture morphology of samples is considered to be an indicator of their properties and may be influenced by viscosity, proportion of components, and obtaining methods. In the case of the E–B and E–Cl–B samples, it can be noticed that there are no distinct domains, the mixtures having a homogeneous structure. The fracture surface of the E–Br–B sample has a roughness aspect with much larger waveform morphology which determines a microstructured appearance similar to the EPDM (E) sample. The micrographs of E–B, E–Cl–B, and E–Br–B composites show the absence of phase separation and cracks, indicating a good compatibility of the components.

**Figure 16:**
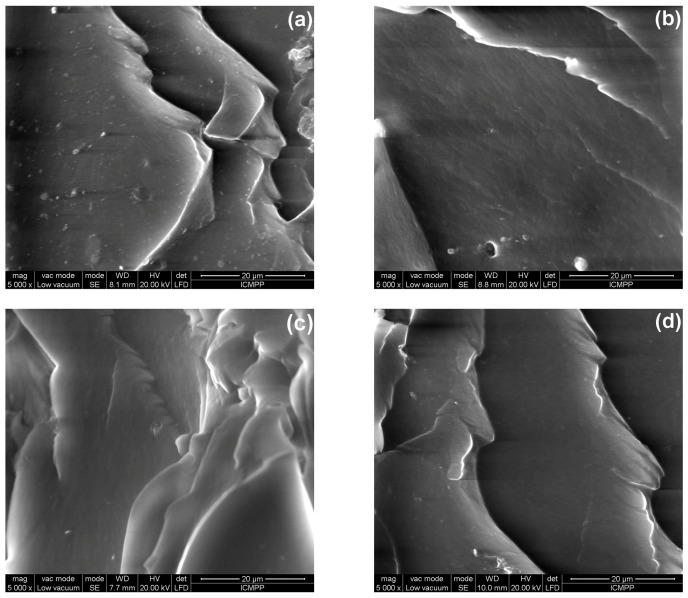
Micrographs of fractured surfaces of EPDM (M (**a**) and EPDM blends (E–B (**b**); E–Cl–B (**c**); E–Br–B (**d**)) after irradiation at 20 kGy (5000× magnification).

In comparison to the morphology of the samples before irradiation, the electron-beam-irradiated samples have relatively smoother morphology (Figure 16). The smoother morphology of systems may reveal an improvement in the compatibility between components of EPDM blends. After irradiation, it can be observed that in the case of E and E–Br–B samples the microdomains have disappeared, leaving a smooth surface with a homogeneous appearance.

### 3.6. Surface Properties

The mean values of contact angle for EPDM/butyl rubber composites determined with water, formamide, and diiodomethane as probe liquids are listed in Table 7. It is found that the contact angle increased after incorporation of butyl rubbers into the EPDM matrix, excepting sample E–B, suggesting the hydrophobic character of the sample surface. In comparison with the nonirradiated sample M, the contact angle for polar solvent drops (water, formamide) of the E–B surface decreases from 92.3° to 87.2° and from 85.9° to 79.5°, respectively (Table 7), indicating that the hydrophilic groups (OH and C=O) are accessible to the surface top. Contrarily, samples E–Cl–B and E–Br–B have registered water contact angle values of 99.5° and 100.1°, more greatly increased than those corresponding to the sample M, which can be caused by the abundance of free methyl groups CH_3_ oriented to the surface. As also shown in Table 7, the values of contact angles for all electron-beam-irradiated samples increase as the irradiation dose increases, maintaining the hydrophobic character of the surface of these composites. This behavior demonstrates a strong coupling of the stabilizer (Irganox 1010) with the elastomers, inducing advanced protection against the oxidation reaction in the irradiated rubber composites [44].

The surface free energy (SFE or γ_lv_^TOT^), dispersion, and polar components of the samples can be calculated using the van Oss–Good approach [45,46,47]:

(8) $$\left(1+\cos\theta \right)\times \gamma_{lv}^{TOT}=2\left(\sqrt{\gamma_{sv}^{LW}\times \gamma_{lv}^{LW}}+\sqrt{\gamma_{sv}^{+}\times \gamma_{lv}^{-}}+\sqrt{\gamma_{sv}^{-}\times \gamma_{lv}^{+}}\right)$$

(9) $$\gamma_{sv}^{AB}=2\sqrt{\gamma_{sv}^{+}\times \gamma_{sv}^{-}}$$

(10) $$\gamma_{lv}^{TOT}=\gamma_{sv}^{LW}+\gamma_{sv}^{AB}$$

where θ is the contact angle; $\gamma_{lv}^{TOT}$ is the liquid’s total surface tension; $\gamma_{lv}^{LW}$ and $\gamma_{sv}^{LW}$ denote the apolar Lifshitz–van der Waals components of the liquid and the solid, respectively; $\gamma_{sv}^{AB}$ represents the polar Lewis acid–base interaction; and $\gamma_{sv}^{+}$ and $\gamma_{lv}^{-}$, or $\gamma_{sv}^{-}$ and $\gamma_{lv}^{+}$ are the Lewis acid and base contributions either of the solid (*s*) or the liquid (*l*) phase as indicated by subscripts. To solve the resulting systems of equations it is necessary to utilize test liquids with known values for $\gamma_{lv}^{TOT}$, $\gamma_{sv}^{LW}$, $\gamma_{sv}^{+}$, and $\gamma_{sv}^{-}$ [47] (Table 8). The subscripts “*lv*” and “*sv*” denote the interfacial liquid–vapor and surface–vapor tensions, respectively, while superscripts “*p*” and “*d*” denote the polar and dispersive components, respectively, of total surface tension, $\gamma_{lv}^{TOT}$.

To obtain the components of surface free energy and the total free surface energy of the studied samples, information about contact angle (Table 7) and the liquids used for contact angle measurements (Table 8) are needed. As can be observed in Table 7 and Table 9, after irradiation the surface free energy and contact angle of the samples have changed, and the greater part of the change takes place when the samples are exposed to an irradiation dose of 20 kGy, with a slight further modification of these parameters as the irradiation dose increases to 150 kGy. The decrease of total surface free energy as the irradiation dose increases can be associated with a decrease of both polar and dispersion components of the surface energy. An exception was the E–Br–B sample, which presented an increase of $\gamma_{lv}^{TOT}$ from 29.93 to 37.93 mN/m due to the increase of the $\gamma_{sv}^{+}$ component from 1.1 mN/m up to 5.6 mN/m, probably because of more peroxyl radicals (formed as the result of the reactions involving molecular oxygen) which promote surface oxidation.

Different values of the surface energy of EPDM have been reported; this may be caused by the different parameters which describe the material, like type and the amount of diene, the content of additives, and crosslinking degree [47]. Moreover, this slight difference can also be attributed to either morphological differences, surface roughness, dissimilar heterogeneities, or a combination of the above-mentioned properties [48]. In our case, the nonirradiated M sample (EPDM) has the highest value of total surface energy (51.55 mN/m), with a more significant contribution of the dispersive Lifshitz–van der Waals component ($\gamma_{sv}^{LW}$ = 38.85) and less polar forces ($\gamma_{sv}^{AB}$ = 12.7). Taking in account that water is neutral and diiodomethane is a nonpolar liquid, it can be affirmed that formamide is most important in the determination of the ratio between the acid and base surface energy components. Because formamide is a very basic liquid, it will interact with the $\gamma_{sv}^{+}$ acid components from the surface sample. All the investigated samples are considered to be bipolar because both $\gamma_{sv}^{+}$ and $\gamma_{sv}^{-}$ polar forces are present to interact with the material, and the resulting values of the electron donor interactions ($\gamma_{sv}^{-}$) which are provided by C–H_3_, C–Cl, C–Br are higher than the electron acceptor interaction ($\gamma_{sv}^{+}$) usually ensured by hydroxyl groups (Table 9). Moreover, from the values of $\gamma_{sv}^{AB}$ (Table 9), the E–Cl–B sample is more polar than E–Br–B (7.32 mN/m compared to 5.02 mN/m) due to the presence of carbon–chlorine bonds in the rubber blend which are more polar than the carbon–bromine bond.

## 4. Conclusions

Radiation processing of some EPDM/butyl rubber composites induces improved chemical stabilization by sample crosslinking up to an irradiation dose of 150 kGy. The effect of electron beam crosslinking on the mechanical, thermal, and morphological characteristics of these systems was discussed. The incorporation of butyl rubber into the EPDM matrix resulted in compatible compositions. Contact angle measurements showed increased hydrophobicity of these composites as the irradiation dose increased. The fastest increase occurs in the range 20–50 kGy. The electron beam irradiation also influenced the total surface free energy by its decrease with increasing irradiation dose. With the increase of the crosslink density, the tensile strength and elongation at break decrease because of the formation of the network structure. As the crosslink density increased, the thermal stability of the irradiated samples also increased. The Charlesby–Pinner approach indicates that within the experimental conditions of our study, a predominantly crosslinking process under electron beam irradiation occurs.

## Figures and Tables

**Table 1:** Formulation of ethylene propylene diene monomer (EPDM)/butyl rubber composites.

Ingredients (Parts Per Hundred Rubber (phr))	Sample Code			
	M	E-B	E-Cl-B	E-Br-B
EPDM (E)	100	95	95	95
Butyl rubber (IIR)	-	5	-	-
Cl-butyl rubber (Cl–IIR)	-	-	5	-
Br-butyl rubber (Br–IIR)	-	-	-	5
Trimethylolpropane trimethacrylate (TMPT)	3	3	3	3
Antioxidant	0.5	0.5	0.5	0.5
Total (phr)	103.5	103.5	103.5	103.5

**Table 2:** Molar attraction constants, *F_i_*, at 25 °C for repeating units from EPDM, EPDM/IIR, EPDM/Cl–IIR, and EPDM/Br–IIR.

Group	*F_i_* (MPa)^1/2^		
	Small	Hoy	Van Krevelen
–CH_3_	438	303	420
–CH_2_–	272	269	280
–CH–	57	176	140
>CH–CH<	266	422	304
>C–C<	190	655	0
–Cl	552	420	471
–Br	696	528	614

**Table 3:** Summary of molar attraction constants for EPDM rubbers.

Sample/Method	Σ*F_i_*		
	Small	Hoy	Van Krevelen
EPDM	4370	4809.0	4924
EPDM/IIR	4576	6591.5	7328
EPDM/Cl–IIR	6747	6884.5	7519
EPDM/Br–IIR	6891	6992.5	7662

**Table 4:** Values of solubility parameters and of polymer–solvent interaction parameters.

Sample/Method	Small		Hoy		Van Krevelen	
	*δ*_p_	*χ*	*δ*_p_	*χ*	*δ*_p_	*χ*
EPDM	14.4874	0.9325	15.9428	0.5590	16.3240	0.4913
EPDM/IIR	15.0084	0.7779	15.0437	0.7682	16.1246	0.4336
EPDM/Cl–IIR	14.1741	1.0367	14.4630	0.9403	15.7960	0.5884
EPDM/Br–IIR	13.1293	1.4452	13.3227	1.3625	14.5983	0.8976

**Table 5:** DSC data of EPDM/butyl rubber composites.

Sample	T_g1_ (°C)	T_m1_ (°C)	ΔH_m1_ (J g^−1^)	T_g2_ (°C)	T_m2_ (°C)	ΔH_m2_ (J g^−1^)	T_cr_ (°C)	ΔH_cr_ (J g^−1^)	*T*_cc_ (°C)	ΔH_cc_ (J g^−1^)
M	−35	45	11.4	−40	49	22.08	13	−20.66	–	–
M–15	−34	53	7.66	−39	45	22.47	19	−20	27 (h_1_)	−3.15 (h_1_)
E–B	−37	48	13.42	−38	44	18.21	16/33	−21.68	–	–
E–B–15	−32	53	8.96	−39	43	16.42	21	−19.35	25 (h_1_)	−2.97 (h_1_)
E–Cl–B	−35	40/53	9.56	−39	41	12.56	−19/18/31	−19	29 (h_1_)	−1.03 (h_1_)
E–Cl–B–15	−33	54	8.95	−35	45	13.3	20	−16.35	27 (h_1_)	−2.49 (h_1_)
E–Br–B	−36	54	8.84	−39	43	12.4	16	−14.1	25 (h_1_)	−1.09 (h_1_)
E–Br–B–15	−37	54	10.24	−41	45	13.51	23	−15.1	26 (h_1_)	−2.05(h_1_)

h_1_—first heating run; h_2_—second heating run; T_g1_—glass transition temperature corresponding to the first heating run; T_g2_—glass transition temperature corresponding to the second heating run; T_m1_—melting temperature corresponding to the first heating run; T_m2_—melting temperature corresponding to the second heating run; T_cr_—crystallization temperature; T_cc_—cold crystallization temperature; ΔH_m1_—enthalpy of the melting profile corresponding to the first heating run; ΔH_m2_—enthalpy of the melting profile corresponding to the second heating run; ΔH_cr_—crystallization enthalpy; ΔH_cc_—cold crystallization enthalpy.

**Table 6:** TGA results for the thermal degradation of EPDM/butyl rubber composites.

Sample	T_5%_ (°C)	T_max_ (°C)	W (%)	T_10%_ (°C)	T_30%_ (°C)	T_50%_ (°C)	R (%)
M	433	472	98.30	445	462	469	1.21
M–15	445	474	98.10	450	466	472	1.40
E–B	418	470	98.33	432	455	467	1.61
E–B–15	422	473	97.02	434	458	469	2.95
E–Cl–B	410	471	98.81	430	455	466	1.13
E–Cl–B–15	415	474	97.61	435	459	469	1.96
E–Br–B	411	472	96.45	429	455	467	3.50
E–Br–B–15	418	474	95.51	432	457	470	4.30

T_max_—temperature corresponding to the maximum rate of decomposition; T_5%_, T_10%_, T_30%_, T_50%_—temperatures corresponding to 5, 10, 30, and 50 wt % mass losses; W—percentage of mass loss corresponding to each stage; R—residual mass at 700 °C.

**Table 7:** Contact angle values (*θ*) between liquid and sample surfaces.

Samples		Contact Angle Values (*θ*)		
		Water	Formamide	Diiodomethane
Initial	M	92.3	94.3	41.5
	E–B	87.2	79.5	48.8
	E–Cl–B	99.5	86.0	47.1
	E–Br–B	100.1	91.7	66.4
20 kGy	M	108.6	95.5	67.2
	E–B	104.5	93.5	75.0
	E–Cl–B	107.8	98.9	62.8
	E–Br–B	109.5	99.4	64.4
150 kGy	M	112.5	96.9	76.4
	E–B	112.2	104.3	92.4
	E–Cl–B	112.8	106.7	72.6
	E–Br–B	110.8	105.6	61.5

**Table 8:** Surface free energy and its component values for the used liquids; all values are expressed in mN/m.

Liquid	$\gamma_{}^{TOT}$	$\gamma_{}^{LW}$	$\gamma_{}^{AB}$	$\gamma_{}^{+}$	$\gamma_{}^{-}$
Water (bidistilled)	72.8	21.8	51	25.5	25.5
Formamide	58.0	39.0	19	2.28	39.6
Diiodomethane	50.8	50.8	0	0	0

**Table 9:** Surface free energy values and components for sample surfaces; all values are expressed in mN/m.

Samples		$\gamma_{sv}^{LW}$	$\gamma_{sv}^{+}$	$\gamma_{sv}^{-}$	$\gamma_{sv}^{AB}$	$\gamma_{lv}^{TOT}$
Initial	M	38.85	8.3802	16.42	23.46	62.31
	E–B	34.94	1.4680	10.94	8.01	42.96
	E–Cl–B	35.88	2.2094	3.87	5.85	41.73
	E–Br–B	24.91	1.1083	5.68	5.02	29.93
20 kGy	M	24.45	1.1683	2.01	3.07	27.52
	E–B	20.13	0.3147	3.30	2.04	22.17
	E–Cl–B	26.97	2.9563	3.70	6.61	33.58
	E–Br–B	26.05	2.6156	2.89	5.50	31.55
150 kGy	M	19.38	0.2836	0.83	0.97	20.35
	E–B	11.66	0.0973	2.69	1.02	12.68
	E–Cl–B	21.43	3.1203	3.62	6.72	28.15
	E–Br–B	27.71	5.6260	4.64	10.22	37.93

θ = contact angle, $\gamma_{lv}^{TOT}$ = solid’s total surface tension, $\gamma_{sv}^{LW}$ = apolar Lifshitz–van der Waals components of the solid, $\gamma_{sv}^{-}$ and $\gamma_{sv}^{+}$ = electron donor (Lewis base) and electron acceptor (Lewis acid) components, $\gamma_{sv}^{AB}$ = polar Lewis acid–base interaction.
